# Structural Studies of Aliphatic Glucosinolate Chain-Elongation Enzymes

**DOI:** 10.3390/antiox10091500

**Published:** 2021-09-21

**Authors:** Vivian Kitainda, Joseph M. Jez

**Affiliations:** Department of Biology, Washington University in St. Louis, St. Louis, MO 63130, USA; k.vivian@wustl.edu

**Keywords:** glucosinolate, glucosinolate biosynthesis, isopropylmalate dehydrogenase (IPMDH), methionine, methylthioalkylmalate synthase (MAMS), specialized metabolism, structural biology

## Abstract

Plants evolved specialized metabolic pathways through gene duplication and functional divergence of enzymes involved in primary metabolism. The results of this process are varied pathways that produce an array of natural products useful to both plants and humans. In plants, glucosinolates are a diverse class of natural products. Glucosinolate function stems from their hydrolysis products, which are responsible for the strong flavors of Brassicales plants, such as mustard, and serve as plant defense molecules by repelling insects, fighting fungal infections, and discouraging herbivory. Additionally, certain hydrolysis products such as isothiocyanates can potentially serve as cancer prevention agents in humans. The breadth of glucosinolate function is a result of its great structural diversity, which comes from the use of aliphatic, aromatic and indole amino acids as precursors and elongation of some side chains by up to nine carbons, which, after the formation of the core glucosinolate structure, can undergo further chemical modifications. Aliphatic methionine-derived glucosinolates are the most abundant form of these compounds. Although both elongation and chemical modification of amino acid side chains are important for aliphatic glucosinolate diversity, its elongation process has not been well described at the molecular level. Here, we summarize new insights on the iterative chain-elongation enzymes methylthioalkylmalate synthase (MAMS) and isopropylmalate dehydrogenase (IPMDH).

## 1. Introduction to the Glucosinolates

**Figure 1:**
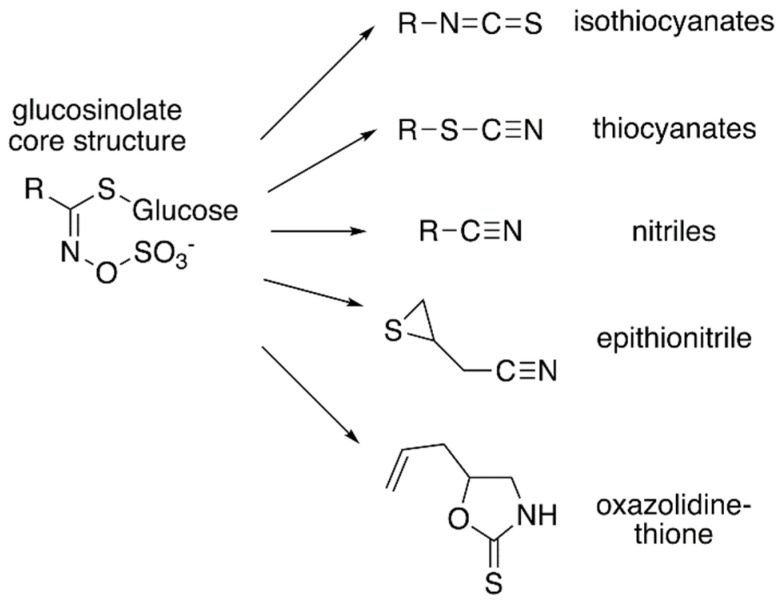
Core glucosinolate structure and hydrolysis product diversity. The core chemical structure of glucosinolates (**left**) consists of a β–D–glucosyl residue linked via a sulfur to a (Z)–N–hydroximinosulfate ester with and a variable amino acid-derived R group. Modifications of the aliphatic, aromatic, or indole R-group leads to the chemical diversity of this class of specialized metabolites. Hydrolysis of various glucosinolates leads to an array of bioactive molecules (**right**), including isothiocyanates, thiocyanates, nitriles, epithionitrile, and oxazolidine-thione.

Glucosinolates are amino acid-derived plant-specialized metabolites that are largely found within the members of the family Brassicaceae, which includes vegetables such as broccoli, cabbage, and mustard, as well as the model plant *Arabidopsis thaliana* (thale cress) [1]. They have been reported in 14 other families from the order Capparales, as well as in the family Euphorbiaceae from the genus *Drypetes*, which is unrelated to other glucosinolate-containing families [2]. Glucosinolates can be classified according to their precursor amino acids. The aliphatic glucosinolates are derived from methionine, alanine, leucine, isoleucine, or valine; aromatic glucosinolates are built from phenylalanine or tyrosine; and the indole glucosinolates originate with tryptophan. Each of class of glucosinolate shares a core chemical structure consisting of a β-D-glucosyl residue linked to a (Z)-N-hydroximinosulfate ester through a sulfur and a variable amino acid-derived R group (Figure 1) [1]. To date, more than 130 glucosinolate molecules, of which *Arabidopsis* contains 40 mainly derived from methionine and tryptophan, have been described [3].

Although the biological activities of all glucosinolates are yet to be elucidated, glucosinolates are believed to serve as responses to a wide range of external and/or environmental stimuli [4,5]. Much of glucosinolate function is related to their hydrolysis products (Figure 1), which accumulate in response to plant tissue damage. The various hydrolysis products are generated by a thioglucoside glucohydrolase known as myrosinase, which hydrolyzes the glucose moiety of the core glucosinolate structure [6,7]. The resulting products are glucose and an unstable aglycone compound that can rearrange to form isothiocyanates, nitriles, or other hydrolysis products, based on the starting glucosinolate structure type (Figure 1). The hydrolysis activity appears to be hindered by the physical separation of glucosinolates and myrosinase in intact plant tissue [1]. Glucosinolate hydrolysis products are involved in communicating a range of information pertaining to plant defense against insects, bacteria, and fungi. Some hydrolysis products, such as isothiocyanates, can be hydrolyzed further by the phenylalanine ammonia lyase to generate toxic compounds that can be injurious to certain pathogens [4]. Hence, some studies have proposed that glucosinolates may have a more direct role in plant defense.

Interest in glucosinolates has long been present in human society, mainly due to the distinct taste and flavors of certain Brassicaceae vegetables (cabbage, cauliflower, broccoli) and condiments (mustard, horseradish, wasabi) that are present in our diet [8,9]. Glucosinolates have gained significance in an agricultural sense with the increased importance of rapeseeds/canola (*Brassica napus*, *B. rapa*, and *B. juncea*) as oil crops worldwide [1]. To increase plant use efficiency, plant breeders have reduced the levels of glucosinolates in Brassica oil crops to allow the seedcake (i.e., the protein-rich residue left after seed processing) to be used as a nutritional supplement in animal feed [10]. This is done to avoid the anti-nutritive effects that glucosinolates can have on animals, as one of the major glucosinolates in canola hydrolyzes to an oxazolidine-2-thione, which causes goiter and has other negative effects in cattle [11]. Glucosinolate-rich plants can also be used as biofumigation agents. For example, post-harvest plant material can be incorporated into soils, where the glucosinolate-containing material can suppress pathogen, nematode, and/or weed growth [12,13,14]. Breeders have also tried to modify glucosinolate levels in rapeseed foliage to address damage from fungal and insect pests [15,16]. For human health, glucosinolates are potentially useful because of their reported cancer-preventative action in animal models. For example, 4-methylsulfinylbutyl glucosinolate, which is found in broccoli, hydrolyzes to the isothiocyanate sulforaphane, a molecule that blocks the cell cycle and promotes apoptosis to fight tumor growth [8,17,18,19,20]. Sulforaphane can also slow effects of *Helicobacter pylori*-caused gastritis and stomach cancer [21]. Overall, glucosinolate engineering in plants and production platforms such as bacteria offer useful tools for the application of these natural products in plant defense, agriculture, human health, and animal nutrition.

## 2. Glucosinolate Biosynthesis

**Figure 2:**
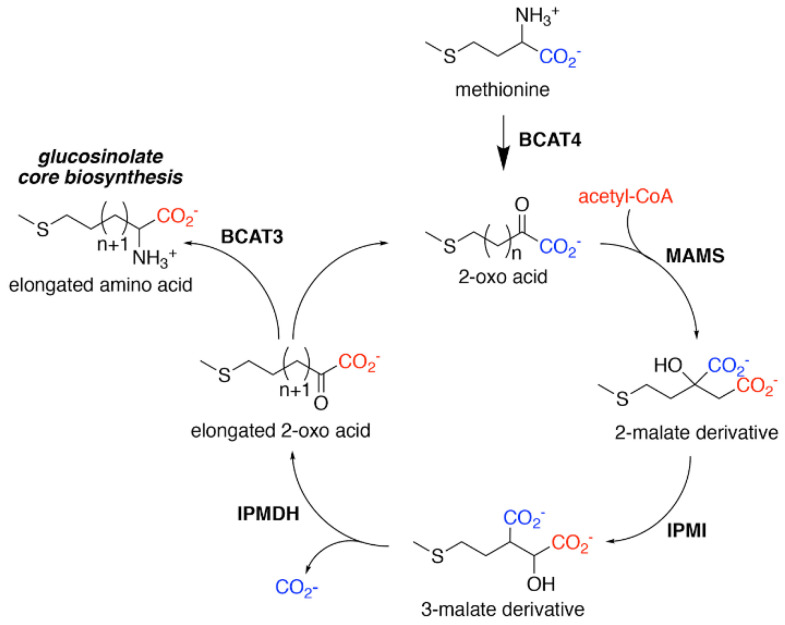
Aliphatic Glucosinolate Chain Elongation. Conversion of methionine to its corresponding 2-oxo acid (4–(methylsulfanyl)–2–oxobutanoate) by branched-chain amino transferase 4 (BCAT4) provides the starting point for the chain elongation cycle. A series of reactions catalyzed by methylthioalkylmalate synthase (MAMS), isopropylmalate isomerase (IPMI), and isopropylmalate dehydrogenase (IPMDH) result in elongation of the aliphatic chain by a single methylene group. The elongated 2–oxo can undergo transamination to the corresponding amino acid by BCAT3 or reenter the chain elongation cycle.

**Figure 3:**
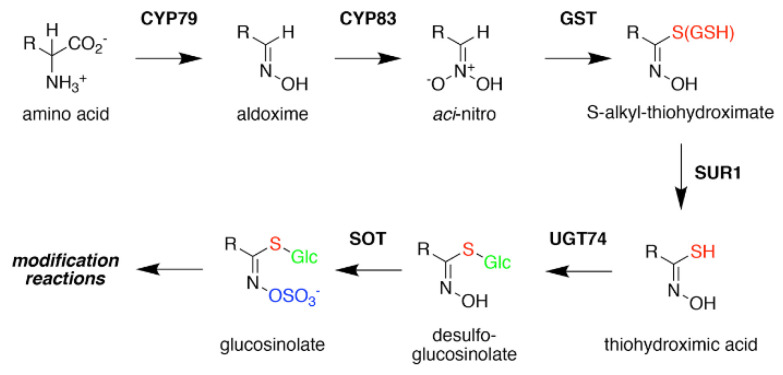
Glucosinolate Core Biosynthesis. A variety of amino acids (i.e., variable R-group), including elongated aliphatic methionine-derived molecules (see Figure 2), can be converted to aldoximes by members of the CYP79 cytochrome P450 family to begin construction of the core glucosinolate scaffold (see Figure 1, left). Members of the CYP83 family generate the unstable aci–nitro compounds believed to be the substrate of glutathione-S-transferases that introduce the shared sulfur atom. The SUR1 cysteine-sulfur lyase converts the S-alkyl-thiohydroximate to thiohydroximic acid, which is then modified with a glucosyl-residue by UDP-glucosyltransferase (UGT) family 74 enzymes. The final step of the pathway involves sulfation by PAPS-dependent sulfotransferases (SOT). Subsequent modification reactions of the core structure yield the molecular diversity of glucosinolates.

Biosynthesis of glucosinolates requires the integration of multiple building blocks to generate their shared chemical structure. For the aliphatic glucosinolates, methionine provides the starting point of their biosynthesis through an iterative series of reactions that elongate the R-group, i.e., aliphatic chain elongation (Figure 2). In the second stage of aliphatic glucosinolate synthesis (Figure 3), the methionine-derived elongation products then undergo a series of modifications, including addition of the sugar and sulfate groups, to yield the core glucosinolate structure. A similar set of reactions for the aromatic and indole glucosinolates builds directly from the initial amino acids. Once the core glucosinolate structure is assembled, the third stage of biosynthesis consists of various secondary modifications to the R-group generate the diverse array of described glucosinolates [22,23,24,25,26,27].

For aliphatic glucosinolate synthesis, the chain-elongation process begins with deamination of methionine to the corresponding 2-oxo acid, which is catalyzed by branched-chain amino acid aminotransferase 4 (BCAT4) (Figure 2) [28,29]. Although BCAT4 is localized in the cytosol, the rest of the enzymes involved in the elongation pathway are localized in the chloroplast [30,31,32,33,34]. This indicates the need for import of 2-oxo acids into the chloroplast, a function that has been potentially assigned to a chloroplast-localized bile acid transporter BAT5 [35]. The next reactions in the sequence require a set of three enzymes—methylthioalkylmalate synthase (MAMS), isopropylmalate isomerase (IPMI), and isopropylmalate dehydrogenase (IPMDH) (Figure 2). These enzymes form an iterative cluster of reactions that elongate the aliphatic chain of the 2-oxo acid. MAMS catalyzes the condensation of the 2-oxo acid and acetyl-CoA to form a 2-malate derivative [30,31,36,37,38]. Isomerization of the 2-malate derivative to a 3-malate derivative is catalyzed by IPMI [33,39]. The final step in the elongation cycle is performed by IPMDH, which oxidatively decarboxylates the 3-malate derivative to a 2-oxo acid, which elongates the original 2-oxo acid by a methylene group [34,40,41]. After each cycle, the elongated 2-oxo acid can either proceed through another round of chain elongation or be transaminated by BCAT3 to yield homomethionine (or its elongated derivatives), which enters the core glucosinolate assembly pathway [42,43] (Figure 2).

Formation of the glucosinolate core structure takes place in the cytosol and involves a set of reactions shared by aliphatic, aromatic, and indole glucosinolates (Figure 3). Conversion of elongated methionine-derived amino acids, along with phenylalanine, tyrosine, and tryptophan, into aldoximes is performed by a set of cytochrome P450s of the CYP79 family. CYP79F1 catalyzes the reaction with all elongated methionine derivatives, while CYP79F2 only converts long-chain methionine derivatives; CYP79B3 catalyzes the reaction with tryptophan; and CYP79A2 uses phenylalanine [44,45,46,47,48,49]. The aldoximes are oxidized to either nitrile oxides or aci-nitro compounds by CYP83 cytochrome P450s. CYP83B1 oxidizes both the tryptophan and phenylalanine-derived acetaldoximes, while CYP83A1 converts various aliphatic aldoximes [50,51,52,53,54].

The resulting cytochrome P450 products are conjugated to the sulfur donor glutathione by glutathione-S-transferases to produce S-alkyl-thiohydroximates, which serve as substrates for the carbon-sulfur lyase SUR1 [55]. This is the first enzymatic transformation in the core glucosinolate biosynthesis pathway that links their assembly to enzymes of sulfur metabolism [7,56]. The thiohydroximates generated by SUR1 undergo S-glycosylation catalyzed by glucosyltransferases of the UGT74 family to form desulfoglucosinolates. UGT74C1 glucosylates the methionine-derived molecules and UGT74B1 modified the aromatic amino acid-derived compounds [57,58].

Sulfation of the desulfoglucosinolates to yield the final glucosinoate molecule is mediated by a set of sulfotransferases (SOTs) that use 3′-phosphoadenosine-5′-phosphosulfate (PAPS) as a sulfate donor [59,60,61,62,63]. This is the second step in the assembly of glucosinolates that depends on sulfur metabolism, as the activity of the adenosine-5′-phosphosulfate kinase is essential for PAPS formation and generation of a wide array of sulfonated metabolites and that PAPS synthesis responds to sulfur nutrient levels in plants [64,65,66,67,68,69,70,71]. Like many of the enzyme families involved in glucosinolate biosynthesis, plants encode multiple SOTs with certain members of the SOT family, such as SOT16, SOT17, and SOT18 in *A. thaliana*, catalyzing the modification of desulfoglucosinolates [60,61,62]. Recent biochemical and X-ray crystallographic studies of Arabidopsis SOT18 identified the active site responsible for the reaction chemistry and key residues required for binding of PAPS; these features are conserved across SOT from plants and other organisms, including humans [72]. Interestingly, the molecular basis of substrate preference of the SOT in glucosinolate biosynthesis remains elusive. For example, comparison of the residues in the SOT active site between Arabidopsis SOT16, which prefers indole desulfoglucosinolates and the methionine-derived desulfoglucosinolate metabolizing SOT17 and SOT18, suggested that the basis of substrate selectivity involves residues beyond the ligand binding site and may be dictated by multiple conformationally flexible loops near the active site [72]. This is a biochemical problem not limited to the plant SOT, but is relevant for SOT families in other organisms, including humans.

Although the various biosynthetic steps in the aliphatic chain-elongation process and the assembly of the core glucosinolate scaffold have been determined, the molecular details for the evolution of these specialized processes and the biochemical selectivity for glucosinolate biosynthesis remain to be determined. Bioengineering efforts for the application of glucosinolates in plant defense, agriculture, human health, and animal nutrition require the knowledge of not only how glucosinolates are synthesized, but also what the specific enzymatic mechanisms and structures of enzymes involved in the glucosinolate biosynthetic pathway are. As a good segment of glucosinolate diversity comes from the elongation step of methionine-derived glucosinolates, the rest of the review will delve into recent structural and biochemical studies concerning enzymes involved in this step. In particular, recent work on MAMS and IPMDH provide insights on the molecular basis for two steps in the chain-elongation cycle of the methionine-derived glucosinolates.

## 3. Aliphatic Chain-Elongation: Methylthioalkylmalate Synthase (MAMS)

The methionine-derived glucosinolates are the most abundant class of glucosinolates in Arabidopsis and Brassicaceae crops, and all share the core elongation reactions that lead to varied side chain lengths [31,37,73,74,75,76,77,78]. In the iterative cycle of reactions that elongate the methionine-derived side chain of aliphatic glucosinolates (Figure 2), MAMS catalyzes the initial condensation of the 2-oxo acid (i.e., 4-(methylsulfanyl)-2-oxobutanoate) with acetyl-CoA to form a 2-malate derivative, as well as subsequent condensation reactions with elongated 2-oxo acids [79].

Phylogenetic analyses suggest that MAMS is evolutionarily related to isopropylmalate synthase (IPMS), which catalyze the first reaction of leucine biosynthesis [37,77]. IPMS and MAMS are members of the DRE-TIM metallolyase superfamily [80]. Sequencing of the MAMS genes in *Arabidopsis* reveals three clades of MAMS (MAMa, MAMb and MAMc) resulting from whole genome duplication and specialization of the MAMS duplicates [76]. In *A. thaliana*, MAMS1 and MAMS2 (MAMa-related) synthesize C_3_-C_5_ aliphatic glucosinolates [36,74,75,76], while MAMS3 (MAMb homolog) produces the shorter chain and C_6_-C_8_ glucosinolates [31]. MAMc was lost in *A. thaliana* but is still found in other *Arabidopsis* species (*A. lyrata*, etc.) [76]. In *B. juncea*, whole genome duplication and hybridization resulted in four MAMS isoforms with MAMS1A and MAMS1B generating C_3_-C_5_ glucosinolates and MAMS2A and MAMS2B only performing a single elongation to form C_3_ glucosinolates [38]. Similar isoforms are found in other Brassica species [38]. From the varied substrate/product profiles of the MAMS isoforms, MAMS serves as a gatekeeper for aliphatic glucosinolate biosynthesis and determines whether products continue through the iterative elongation cycle or proceed to the synthesis of the core glucosinolate structure. Recent crystallographic studies of MAM1A from *B. juncea* provide the first three-dimensional views of MAMS and suggest how the active site catalyzes the condensation of acetyl-CoA to 2-oxo acid substrates of varying lengths for iterative biosynthesis [38].

**Figure 4:**
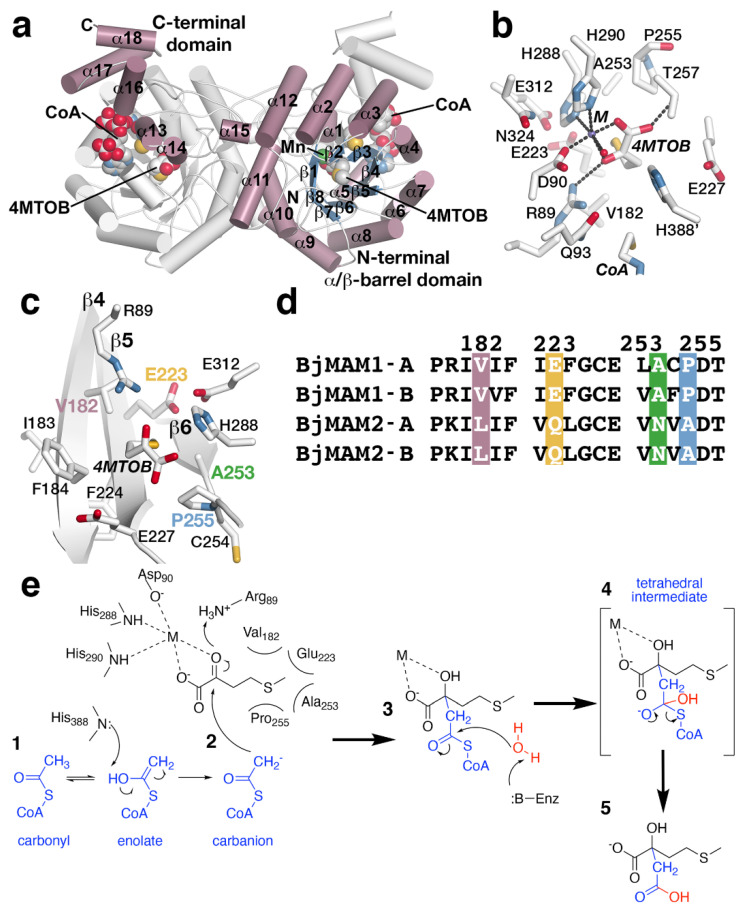
MAMS structure and mechanism. (**a**) Dimeric structure of *B. juncea* MAMS. Respective monomers are colored white and rose with secondary structure features labeled in the rose monomer. Positions of CoA and substrate 4–methylthio–2–oxobutanoic acid (4MTOB) are indicated. (**b**) Active site view showing the location of 4MTOB, divalent metal (M), and CoA. (**c**) View of the substrate binding site. This view centers of the bound 4MTOB and shows the position of the three β-strands that form the back-wall of the site. The four residues (Val182, Glu223, Ala253, and Pro255) that guide substrate preference are indicated. (**d**) Targeted sequence alignment of BjMAM1A/B and BjMAM2A/B. The four residues that dictate substrate preference are indicated. (**e**) Proposed reaction sequence of MAMS-catalyzed condensation of acetyl-CoA to a 2-oxo substrate.

The X-ray crystal structure of MAMS in a dead-end complex with CoA and the substrate 4-methylthio-2-oxobutanoic acid (4MTOB) reveals a dimeric protein with each monomer folded into an N-terminal α/β-barrel domain and C-terminal α-helical domain (Figure 4a). The 2-oxo acid substrate 4MTOB binds in the N-terminal domain with the CoA interacting with both domains. The C-terminal domain of one monomer forms part of the CoA binding site of the adjacent monomer. The location of CoA, a metal ion (Mn^2+^), and 4MTOB define the location of the active site (Figure 4b). Extending into the active site from the surface of the protein, the pantothenate arm of bound CoA places the reactive thiol, which would present the acetyl group for the condensation reaction, in proximity of the C2-carbonyl of 4MTOB and near Gln93 and His388 (Figure 4b). Extensive charge–charge and ionic interactions, which are not shown in the figure, along the length of the ligand lock CoA into the active site. The divalent metal binding site is formed by His288, His290, Asp90, a water molecule, as well as the substrate (Figure 4b). The metal ion helps orient 4MTOB to form additional hydrogen bonds with Arg89 and Thr257 and to position the extended side chain of the ligand toward the interior of the pocket and residues in three β-strands (β4, β5, and β6) (Figure 4c). In this orientation, the aliphatic side chain of 4MTOB forms van der Waals contacts with residues from each β-strand to form a substrate binding pocket (Figure 4c) and contributes to substrate preference.

Given the different substrate/product profiles of the two sets of MAMS in *B. juncea*, comparison of the residues forming the substrate binding sites of the BjMAM1A/B, that catalyze extension of longer chain substrates with the BjMAM2A/B that only accept 4MTOB, highlights replacement of Val182, Glu223, Ala253, and Pro255 in BjMAM1A/B by leucine, glutamine, asparagine, and alanine, respectively, in BjMAM2A/B (Figure 4d). Site-directed mutagenesis was used to test the contribution of each of these residues individually and as a quadruple mutant on substrate preference [38]. Biochemical analysis of the resulting mutants indicated that point mutations did not drastically alter substrate preference and that only the combination of four substitutions changed short- vs. long-chain selectivity [38].

The three-dimensional structure of MAMS suggests a chemical mechanism for the condensation of acetyl-CoA to 2-oxo acid substrates; however, biochemical studies examining the proposed reaction remain to be performed (Figure 4e). In the reaction, the divalent metal, which is coordinated by Asp90, His288, and His290, is essential for activity and interacts with the carboxylate and C2-carbonyl groups of 4MTOB (Figure 4b,c). This interaction orients the aliphatic side chain toward Val182, Glu223, Ala253, and Pro255. Activation of the acetyl-group of CoA is required for addition to the 2-oxo acid (Figure 4e, step 1). This could be achieved by tautomerization of the acetyl-group carbonyl to an enolate, which would allow His388 as a general base to facilitate carbanion formation for the condensation step. Nucleophilic attack of the resulting carbanion on the C2-carbonyl of the 2-oxo acid substrate with Arg89 as a potential general acid (Figure 4e, step 2) leads to in condensation of the acetyl-group with 4MTOB. A water activated by an unidentified general base in the active site likely serves as a nucleophile to react with the thioester carbonyl (Figure 4e, step 3). Glu227 and His388 are possible candidates for this role. The resulting tetrahedral intermediate (Figure 4e, step 4) collapses to a release of free CoA and the extended 2-malate derivative from the active site (Figure 4e, step 5).

The X-ray crystal structure of MAMS also highlights the loss of features from the ‘parent’ IPMS. For example, IPMS is feedback regulated by leucine, which binds to a C-terminal regulatory domain to alter enzymatic activity [81,82]. During evolution of MAMS, the regulatory domain was lost, as the genes encoding MAMS proteins do not contain the corresponding sequence [37]. There are also the loss residues from the N-terminal region of IPMS and changes in the substrate binding site, noted above, that alter the MAMS active site cavity for recognition of 2-oxo acid substrates of varying aliphatic chain length versus the smaller substrate of IPMS [37].

Although MAMS catalyzes the condensation reaction in the elongation pathway, each of the downstream enzymes must also allow binding of substrates of varied length to allow for a complete turn of the synthesis cycle (Figure 2). This raises a question about the biochemical activities of the other enzymes in the elongation cycle because each protein of the pathway could potentially limit the number of elongations. For example, if MAMS produces an elongated substrate that is not accepted by downstream enzymes, then the cycle ends. More structural and biochemical data on the biochemical properties of all the steps in the elongation pathway are needed.

## 4. Aliphatic Chain-Elongation: Isopropylmalate Dehydrogenase (IPMDH)

In aliphatic glucosinolate biosynthesis, IPMDH catalyzes the oxidative decarboxylation of a 3-malate derivative to a 2-oxo acid (Figure 2), a reaction analogous to the enzyme’s role in leucine biosynthesis. Plants encode multiple isoforms of IPMDH. For example, *A. thaliana* has three IPMDH isoforms (AtIPMDH1-3), which differ in biochemical properties and metabolic contribution even with the overall conservation of active site residues between these isoforms [40,41]. AtIPMDH1, despite being able to complement *E. coli* and yeast leucine-auxotrophic mutants, functions primarily in the elongation step of C_4_-C_8_ glucosinolate synthesis, while AtIPMDH2 and AtIPMDH3 are dedicated to leucine biosynthesis [40,41,83,84,85]. The plant IPMDH display high amino acid sequence homology with other bacterial and yeast IPMDHs, all of which belong to the nicotinamide cofactor (NAD(H))-dependent β-hydroxyacid oxidative decarboxylase family [86]. The three-dimensional structure of Arabidopsis IPMDH2, which functions in leucine biosynthesis, and comparisons with the glucosinolate biosynthetic isoform AtIPMDH1 provide insight on the reaction chemistry and residues important for biosynthetic functionalization [40,41,87].

**Figure 5:**
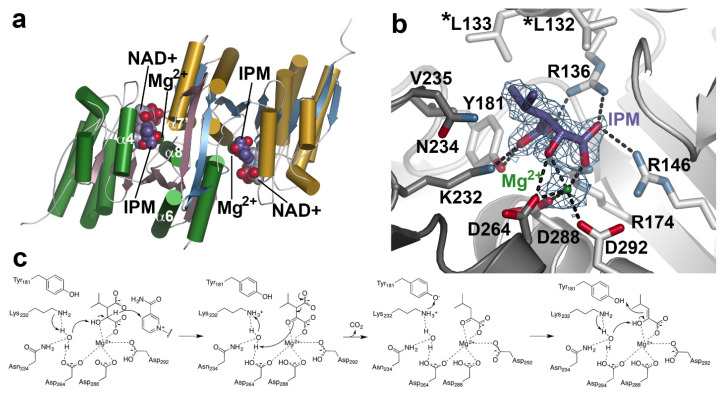
IPMDH Structure and Mechanism. (**a**) Overall structure of AtIPMDH2. The dimeric structure is shown with each monomer colored with green α–helices and rose β–strands or cold α-helices and blue β–strands. Positions of isopropylmalate (IPM), NAD^+^, and Mg^2+^ are shown. (**b**) Active site view. The 2F_o_–F_c_ omit map (1.5 σ) for IPM (purple) and Mg^2+^ (green) is shown. Dotted lines indicate charge–charge and hydrogen bond interactions. The two leucine residues located near the aliphatic side chain of IPM are indicated by asterisks. (**c**) Proposed reaction mechanism of IPMDH.

X-ray crystal structures of AtIPMDH2 in various forms (apoenzyme, isopropylmalate/IPM•Mg^2+^ complex; and NAD^+^ complex) reveal similarities with the bacterial homologs, including function as a dimer, structural changes during binding of nicotinamide cofactor, and conserved active site residues [41,85]. Each monomer in the dimeric structure displays an α/β-architecture with a core anti-parallel β-sheet encompassed by two distinct sets of α-helices (Figure 5a). Within the active site, extensive interactions bind the 3-malate substrate, along with a catalytically required divalent metal ion (Mg^2+^) for catalysis (Figure 5b). The metal ion is coordinated by residues from both monomers (Asp288 and Asp292 from one monomer and Asp264 from the adjacent monomer), as well as the α-carboxylate and β-hydroxyl groups of IPM. In turn, multiple charge–charge and hydrogen bond interactions anchor the substrate in the active site. Across the IPMDH from plants and microbes, the residues forming the catalytic core, IPM binding site, and NAD(H) binding site are highly conserved and allow for a shared reaction chemistry for either leucine or aliphatic glucosinolate biosynthesis [41,87].

The three-dimensional structures of AtIPMDH2 and extensive biochemical analysis of site-directed mutants suggest a reaction mechanism for IPMDH (Figure 5c) [87]. After formation of the E•IPM•Mg^2+^•NAD^+^ complex, Lys232 abstracts a proton from bound water to activate its function as a general base. The activated water accepts a proton from the substrate hydroxyl group with transfer of a hydride to NAD^+^. Next, the Mg^2+^ ion serves as a Lewis acid that facilitates decarboxylation of the β-keto acid. The resulting enol tautomerizes to the ketone product. Importantly, the reaction chemistry is conserved whether the substrate is IPM in leucine biosynthesis or the 3-malate derivative in the aliphatic glucosinolate elongation cycle. These substrates differ in the aliphatic chain length.

Although the three-dimensional structure of an IPMDH with a preference for glucosinolate substrates remains to be determined, sequence comparisons between AtIPMDH1 (glucosinolate biosynthesis) and AtIPMDH2 (leucine biosynthesis) identified a key determinant of substrate preference [41]. The leucine biosynthetic IPMDH retain a pair of leucine residues (Leu132 and Leu 133 in AtIPMDH2; Figure 5b) positioned near the aliphatic chain of the bound substrate; the glucosinolate biosynthetic isoform contains a phenylalanine at the second position (i.e., L133P). Subsequent site-directed mutagenesis and in vitro kinetic and in vivo metabolic analyses confirmed that this change altered substrate preference and led to the evolution of the glucosinolate-specific IPMDH in Arabidopsis [40,41]; however, the structural basis for the substrate selectivity remains to be determined.

## 5. Summary

Glucosinolate biosynthesis has long fascinated plant biologists and chemists. Although these molecules share a basic chemical structure, the range of compounds and their resulting hydrolysis products widely varies. That complexity begins with a set of reactions that evolved from primary metabolism and required subtle changes allowing for new substrate use. Here, we summarized insights from structural studies on two of the enzymes from the metabolic cycle that elongates the aliphatic side chain of methionine-derived glucosinolates. Structural and biochemical studies of MAMS and IPMDH highlight how either a set of active site changes or a single point mutation, respectively, can tailor a protein from primary metabolism for specialized metabolism. Obtaining a similar molecular understanding of the structure and function of the BCATs and IPMI involved in the aliphatic chain elongation of methionine-derived glucosinolates is a logical next step, as it is the combination of all the enzymes in the elongation cycle that leads to the product profiles found in various plants.
